# Complementary SEM-AFM of Swelling Bi-Fe-O Film on HOPG Substrate

**DOI:** 10.3390/ma13102402

**Published:** 2020-05-23

**Authors:** Dinara Sobola, Shikhgasan Ramazanov, Martin Konečný, Farid Orudzhev, Pavel Kaspar, Nikola Papež, Alexandr Knápek, Michal Potoček

**Affiliations:** 1 Department of Physics, Faculty of Electrical Engineering and Communication, Brno University of Technology, Technická 2848/8, 616 00 Brno, Czech Republic; kasparp@feec.vutbr.cz (P.K.); nikola.papez@vutbr.cz (N.P.); 2 Central European Institute of Technology BUT, Purkyňova 123, 612 00 Brno, Czech Republic; martin.konecny@ceitec.vutbr.cz (M.K.); michal.potocek@ceitec.vutbr.cz (M.P.); 3 Faculty of Physics, Dagestan State University, 367015 Makhachkala, st. M. Gadjieva 43-a, Dagestan Republic, Russia; ramazanv@mail.ru (S.R.); farid-stkha@mail.ru (F.O.); 4 Faculty of Mechanical Engineering, Institute of Physical Engineering, Brno University of Technology, Technická 2896/2, 616 69 Brno, Czech Republic; 5 Institute of Scientific Instruments of the Czech Academy of Sciences, Královopolská 147, 612 64 Brno, Czech Republic; knapek@isibrno.cz

**Keywords:** surface delamination, graphite substrate, atomic layer deposition, combined imaging, surface tension

## Abstract

The objective of this work is to study the delamination of bismuth ferrite prepared by atomic layer deposition on highly oriented pyrolytic graphite (HOPG) substrate. The samples’ structures and compositions are provided by XPS, secondary ion mass spectrometry (SIMS) and Raman spectroscopy. The resulting films demonstrate buckling and delamination from the substrates. The composition inside the resulting bubbles is in a gaseous state. It contains the reaction products captured on the surface during the deposition of the film. The topography of Bi-Fe-O thin films was studied in vacuum and under atmospheric conditions using simultaneous SEM and atomic force microscopy (AFM). Besides complementary advanced imaging, a correlative SEM-AFM analysis provides the possibility of testing the mechanical properties by using a variation of pressure. In this work, the possibility of studying the surface tension of the thin films using a joint SEM-AFM analysis is shown.

## 1. Introduction

The combination of bismuth ferrite (BFO) and highly oriented pyrolytic graphite (HOPG) has potential in the preparation of thin films with a magnetoelectric effect and an electrode in the form of multi-layer graphene [1,2]. The symmetry of bismuth ferrite allows the existence of a linear magnetoelectric effect. Bismuth ferrite is a G-type antiferromagnet and exhibits net magnetization when the film’s thickness is below 62 nm [3]. The observation of the magnetization in bulk samples is impossible because of the presence of a spatially modulated spin structure. The destruction of this feature can be achieved by both the replacement of bismuth ions by isovalent cations and the preparation of thin films [4,5,6]. Atomic layer deposition (ALD) was reported to be a reliable method for BFO deposition on silicon oxide/platinum (from Ferrocene (Fe(Cp)_2_) and Bi-triphenyl (Bi(ph)_3_) precursors) [7,8], on zinc oxide (from Bismuth (III) 2,3-dimethyl-2-butoxide and iron (III) tert-butoxide precursors) [9], silicon (from β-diketonate, tris (2,2,6,6-tetramethyl-3,5-heptanedionato) iron(III) (Fe(TMHD)_3_) and Bi(TMHD)_3_ precursors) [10] and strontium titanate (from Bi(thd)_3_ and Fe(thd)_3_ precursors) [11]. Besides the variation in precursors, ALD methods for BFO differ by the combination of cycles [12,13], possible plasma enhancement of the deposition [14], different temperatures of the process [15] and post-treatment [16].

Swelling and bubble formation can be both an undesirable result of the film production process and an intentional surface modification, for example, by laser irradiation [17]. The formation of swelling and bubbles is possible both in heterostructures and in materials of the same chemical composition.

The focus of this work is on using simultaneous scanning electron microscopy (SEM) and atomic force microscopy (AFM) for the description of delamination areas. SEM and AFM are the most popular methods in material science for the study of surface appearance. They allow the diagnostics and investigation of the surface and near-surface area in high resolution. In the case of correlative SEM-AFM microscopy, SEM provides both imaging and navigation through the region of interest. The same sample can be subjected to AFM measurement for nanoscale data acquisition on the surface height. Installing AFM in SEM has several advantages, protecting the device from acoustic vibrations, thermal drift and other noise exposure in the chamber of an electron microscope.

The investigation of delaminations is of interest to both practical applications and the detection of rare physical effects in this structure. In the near-surface region of the substrate, significant residual stresses arise due to the formation of highly nonequilibrium nanostructured and nano-phase states. The patterns of formation of such states are not well studied yet.

## 2. Materials and Methods

### 2.1. Samples Preparation

A commercial sample of highly ordered pyrolytic graphite (HOPG), 7 mm × 7 mm, ZYA grade was used. The samples were purchased from NT-MDT Spectrum Instruments (Zelenograd, Russia). The processing of the substrate material is important for the preparation of heterostructures [18]. The upper layers of the HOPG were mechanically exfoliated using 3M adhesive tape, and the freshly prepared sample was immediately placed via a vacuum feedthrough into a high vacuum chamber with a residual gas pressure of 10^−7^ Pa.

An ALD set-up ALDCERAM ML-200 (ALDCERAM, LLC, Boulder, CO, USA) was used for the preparation of Bi-Fe-O films. To obtain the Bi-Fe-O composition, Bi(mmp)_3_ (mmp = 1-methoxy-2-methyl-2-propanolate, Sigma-Aldrich spol. s r.o., Prague, Czech Republic) was used as the source of Bi. Ferrocene (Fe(C_5_H_5_)_2_, Sigma-Aldrich spol. s r.o., Prague, Czech Republic) realized the Fe component. In the time interval between letting the precursor into the chamber, ozone was supplied for 4 s. A purge was carried out with N_2_ carrier gas of 99.999% purity.

The evaporation temperature of Bi(mmp)_3_ was in the interval of 408–418 K. It provided sufficiently reproducible impulses of precursors. The optimum temperature for the evaporation of the ferrocene precursor was 364 K [19]. The reason was that Bi (mmp)_3_ provided a higher growth rate than Fe(C_5_H_5_)_2_. The growth rate was ~0.12 nm/super cycle. For the homogeneous mixing of the resulting Bi-Fe-O layer, the layer growth of BiO_x_ and FeO_x_ compounds was carried out in a single technological cycle. The initial layer was 150 cycles of BiO_x_, with a super cycle of Bi(mmp)_3_ input at 1.2 s. Then, a layer of FeO_x_ was deposited during 150 cycles. The super cycles of the intake of Fe(C_5_H_5_)_2_ took 2 s each. The inlet gas pipelines transporting the precursors were at 423 K [19]. The substrate was placed 4 cm from the inlet. The chamber was heated uniformly to 523 K and the outlet gas was kept at a constant temperature of 423 K.

### 2.2. Characterization Techniques

A qualitative analysis of the samples in depth was carried out by secondary ion mass spectrometry (SIMS) using a TOF-SIMS^5^ set-up (IONTOF, Muenster, Germany). Depth profiling was performed using a Bi^+^ 30 kV analysis beam of 200 μm^2^ area and O_2_^+^ 500 V for sputtering. The O_2_^+^ beam also had a signal intensity enhancing effect. Raman spectroscopy was carried out on a confocal Raman imaging system Alpha 300 R (WITec, Ulm, Germany) using a 5 mW and 532 nm irradiation laser. The additional study of the surface composition was carried out by an AXIS SupraTM X-ray photoelectron spectrometer (XPS) (Kratos Analytical Ltd, Manchester, UK). A cluster source of argon ions was used for depth profiling with the following characteristics: 5 keV Ar500+, 2 mm × 2 mm etching area, 5 s of pre-etching and post etching time, 1800 s etching time of one circle. The reason for the low energy and long time of etching was the prevention of the preferential etching of oxygen. The data were processed by CasaXPS v.2.3.23 software (Casa Software Ltd, Wilmslow, UK).

A correlative SEM-AFM analysis was carried out using a scanning probe microscope. LiteScope^TM^ (NenoVision, Brno, Czech Republic) integrated to SEM Lyra 3 (Tescan, Brno, Czech Republic). Topography detection was, in this case, performed by self-sensing tuning fork probes which detected the change in their resonance properties due to the change in distance to the sample. The chamber pressure during measurement was 10^−2^ Pa. When measured in air, the pressure was normal and equal to 101,325 Pa. The scanning of the topography at atmospheric pressure was carried out immediately after scanning in vacuum. This allowed avoiding excessive pollution from outside and accidental jumps to another area, which could be the result of mechanical manipulations.

## 3. Results

### 3.1. Samples Comosition

**Figure 1:**
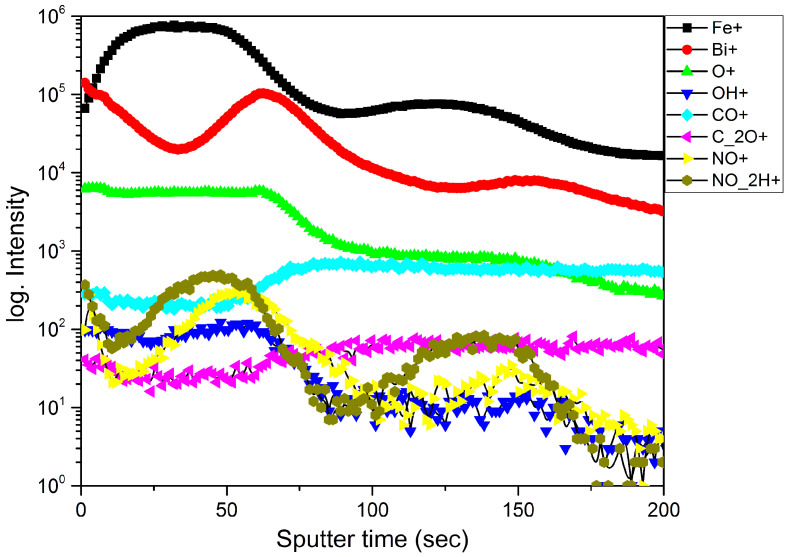
Secondary ion mass spectrometry (SIMS) surface component distribution profile.

Figure 1 shows increased the bismuth content at the surface according to the SIMS analysis. The bismuth atom is relatively large and has the ability to concentrate on surface defects. The incorporation of bismuth into the defective surface layer of graphite leads to the breaking of weak Van der Waals bonds. The resulting stress in the film leads to the appearance of cracks and peeling. The gaseous reaction products formed during sputtering are caught in the surface layers of graphite, causing swelling. SIMS confirms the distribution of elements on the surface associated with the redistribution and self-organization of elements due to delamination of the substrate.

Oxygen is distributed evenly over the thickness of the film; it is a part of iron and bismuth oxides and is also in the combination of gaseous products. The bismuth concentration is higher on the surface and near the substrate, i.e., on parts of the film with the greatest number of defects and dangling bonds. In addition, the presence of a small amount of OH groups was observed. This is because ozone decomposes at a temperature of 250 °C, with a half-life of 1.5 s (O_3_ = O_2_ + O*). According to the literature [20], a redox reaction occurs on the surface, resulting in the formation of surface hydroxyl groups (OH -) due to the transfer of hydride (H-) from organic ligands to adsorbed O atoms. However, at sufficient concentration and temperature, surface hydroxyl groups can partially bind due to diffusion, leaving active oxygen centers on the surface. These secondary ions can also appear as a result of the interaction of an ionizing oxygen beam with the residual hydrogen in the film, since the presence of hydrogen in ALD films is possible [21]. According to the SIMS depth profile, nitrogen-containing secondary ions (NO_2_H^+^ and NO^+^) are present in the film but at a very low concentration in comparison with the main components. We suppose that there are two possible reasons for the appearance of nitrogen in the film. The first reason is the impurities that can enter the reaction chamber through the ozonizer. Oxygen of 99.2% purity which contains residual nitrogen was used. Thus, at the stage of ozonation, the oxidized nitrogen could get into the chamber according to Equation (1):

N_2_ + 4O_2_ + hv = 2NO + 2O_3_ (inside the ozonizer). (1)

Then, Equations (2) and (3) are possible inside the chamber:

NO + O_3_ = NO_2_ + O_2_ (inside the chamber), (2)

NO_2_ + O* = NO + O_2_(inside the chamber). (3)

These reaction products can be captured by surface states or defects, causing even more stratification of the surface. The second reason is connected with the fact that the nitrogen-containing components, as can be seen from the SIMS profile, are mainly concentrated at the same depth as the iron. This allows suggesting that iron can catalyze a nitrogen fixation reaction, similar to reports in literature [22,23]. The values of the nitrogen concentration (Figure 1) in the resulting Bi-Fe-O film are ~3 orders less than then the values of the metal phases. The secondary ions CO^+^ and C_2_O^+^ appear in the depth below Fe and Bi and can be associated with the oxidation of carbon by the O_2_^+^ ionizing beam.

The bismuth surfactant properties contributed to the retention of gas molecules under the surface of the bubbles and the initial adhesion of the reaction products on the surface of the substrates. It can be assumed that the initial vacuum of the ALD process (in the order of 10^−1^ Pa) as well as small amount of impurities in the reaction gases and precursors can be the reason for the rest of the latter in the growing film.

**Figure 2:**
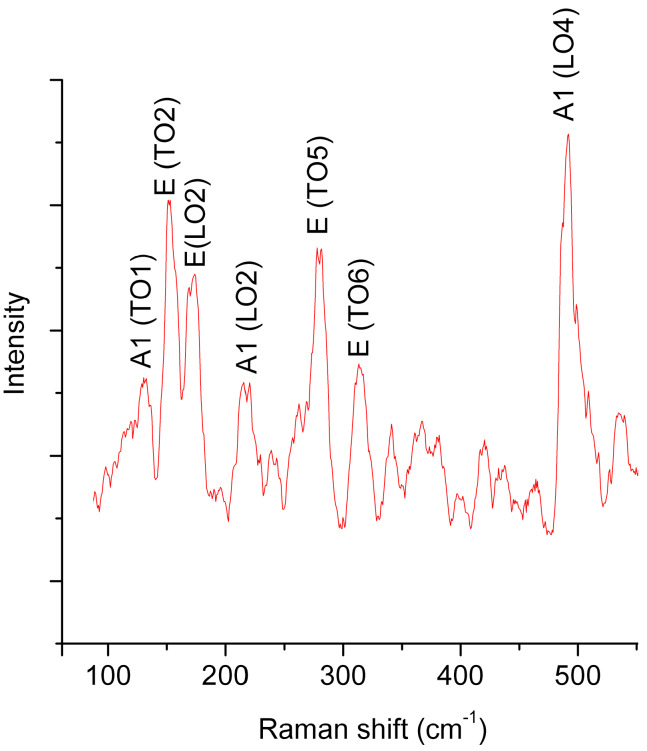
Raman spectra of the self-organized Bi-Fe-O film at the highly oriented pyrolytic graphite (HOPG) substrate.

Raman spectroscopy was performed to determine the molecular bonds in the Bi-Fe-O system. The Raman spectrum contains A1(TO1) 133 cm^−1^, E(TO2) 153 cm^−1^, E(LO2) 172 cm^−1^, A1(LO2) 216 cm^−1^, E(TO5) 280 cm^−1^, E(TO6) 314 cm^−1^ and A1(LO4) 489 cm^−1^ phonons (Figure 2).

These LO and TO phonons indicate the presence of self-organized bismuth ferrite [24,25].

**Figure 3:**
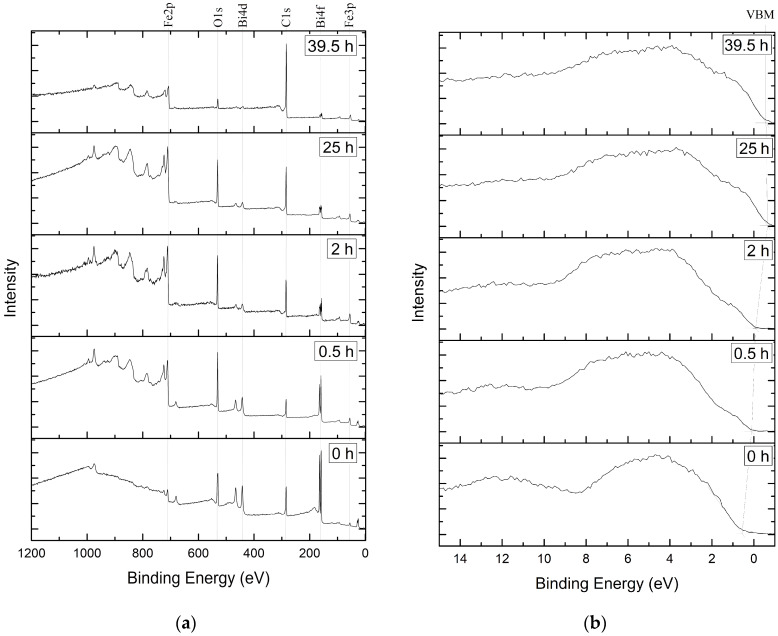
XPS survey spectra of the etched surface (**a**) and valence band spectra (**b**).

XPS survey spectra (Figure 3a) and valence band spectra (Figure 3b) show a significant change in the elements’ distribution [26,27,28] and oxygen chemical state during depth profiling. The variation in the valence band maxima (VBM) is derived from the O2p states [29]. The XPS data were calibrated to a carbon peak at 284.8 eV.

**Figure 4:**
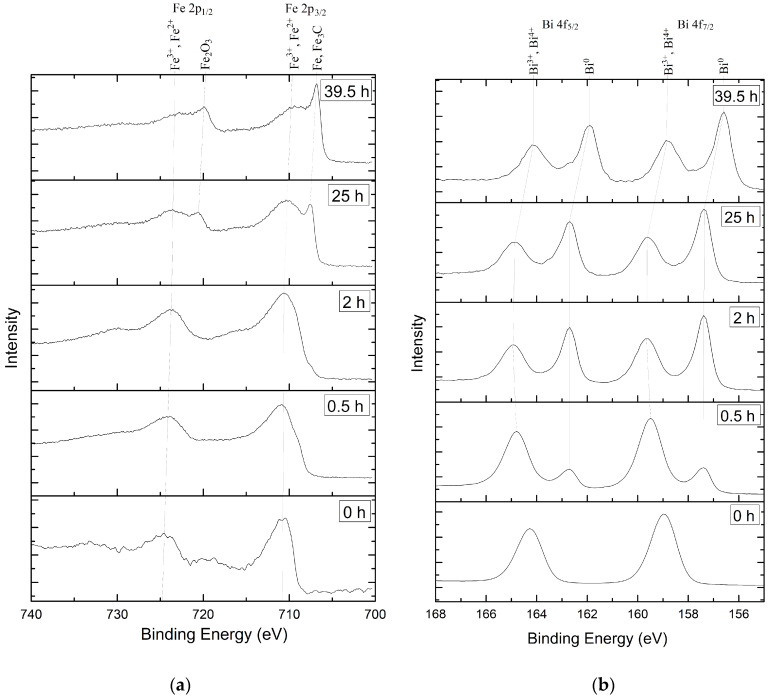
XPS element spectra of the etched surface: (**a**) Fe2p; (**b**) Bi4f.

The Fe 2p_3/2_ peak shows the Fe^3+^ state at 710.8–710.6 eV at the surface and near-surface area (Figure 4a). The difference between the Fe 2p_3/2_ and Fe 2p_1/2_ peak of about 14 eV is evidence of the presence of the Fe^3+^ oxidation state [30]. The chemical state of Fe^2+^ appears after 39.5 h of etching at 709.4 eV [31]. The peaks at 707.5 eV and 706.8 eV correspond to Fe-C and pure Fe [32]. They occur after 20 h of etching and can be clearly observed in Figure 4a after 25 h of etching. Even after 39.5 h of etching, the characteristic Fe^3+^ peak could be observed at the 719 eV and 720 eV satellite peaks. The surface before profiling is converted by Bi^3+^ and shows the XPS peaks of Bi 4f_7/2_ and Bi 4f_5/2_ at 158.9 eV and 164.3 eV [33]. The shift in the binding energy to lower energies for the Bi4f spectra is connected with the presence of defects and amorphization of bismuth closer to the HOPG substrate (Figure 4b). The increasing number of free electrons caused by broken Van der Waals bonds also contribute to the low-energy shift of the spectra due to the increasing length of the Bi-O bond. The spectra displacement after long-time etching to smaller binding energies also indicates the decreasing interaction between the bismuth oxide and the oxidized graphite surface [34]. The etching over 39.5 h reveals bismuth peaks at 164.08 eV and 158.78 eV, which are assigned to bismuth subcarbonate composition [35].

**Figure 5:**
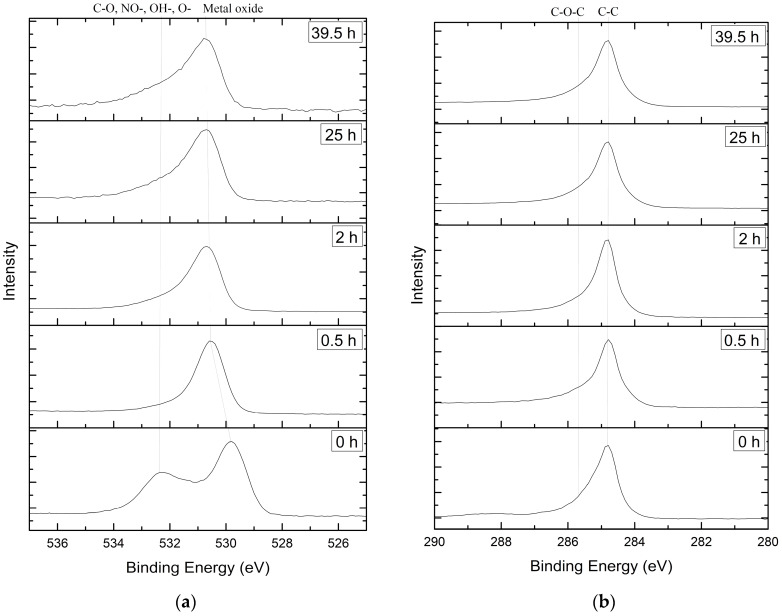
XPS element spectra of the etched surface: (**a**) O1s; (**b**) C1s.

According to Jaiswar Sh. and Mandal K.D. [36], O1s XPS peaks at a lower binding energy (529–530.7 eV) are attributed to lattice oxygen (Figure 5a). Carbon oxide on the surface of the samples is reflected by the O1s XPS peak at 532 eV. After the etching of the film, this peak appears as a part of the shoulder at 531–532 eV. The spectra shoulders around 531 eV refer to excess oxygen (marked as O^−^) [37]. The incorporated nitro (532.7 eV) [38] and hydroxyl (533 eV) [39] groups cause a shift in O1s and are responsible for the high-energy shoulder. Besides the calibration of the C-C peak, the C-O-C bond can be observed at 286 eV at the shoulder of the C1s peak (Figure 5b).

### 3.2. Correlative SEM-AFM

**Figure 6:**
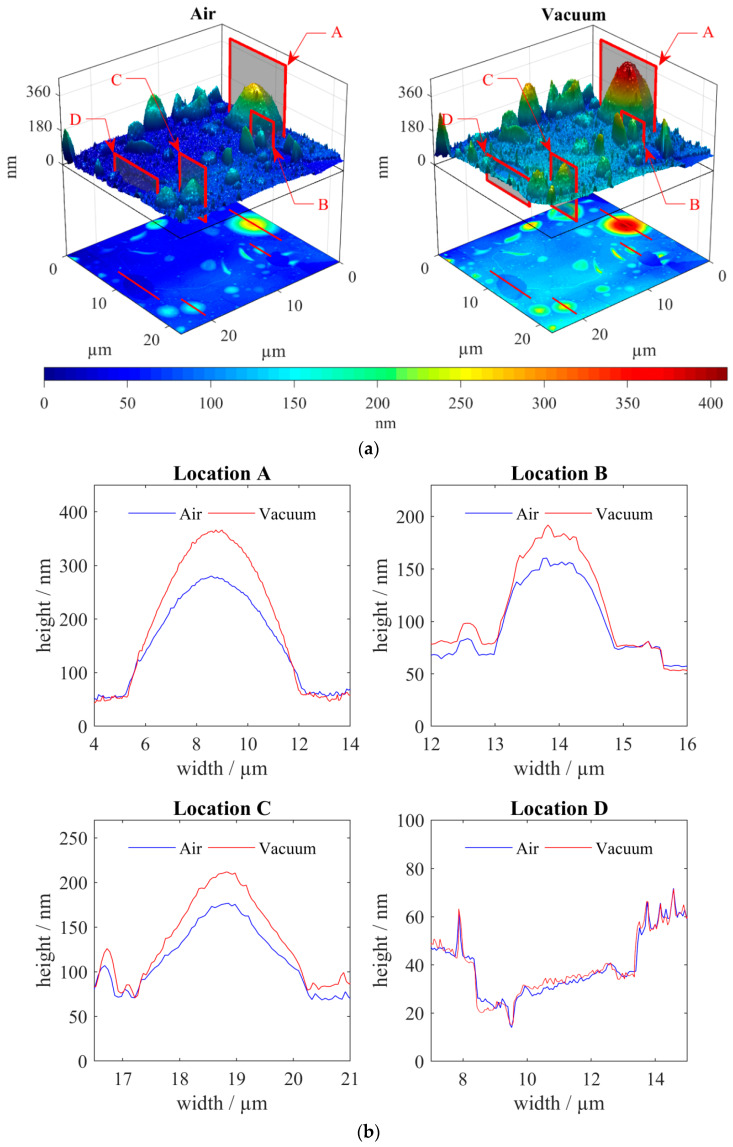
The image of the swollen part: (**a**) 3D atomic force microscopy (AFM) scan in vacuum and in air; (**b**) comparative profiles of the height of the bubbles and holes.

Correlative SEM-AFM allows the fast and easy comparison of images from both techniques. The localization of the region of interest using SEM allows one to quickly focus on the surface of the sample by selecting the desired scan area (Figure 6a). The height of the “bubbles” changed at vacuum and was greater than in air. This is due to the high gas content in them. To confirm the reliability of the results, the depth of the craters—formed at the places where the film was no longer—was measured. It remained virtually unchanged both in vacuum and at normal pressure. The difference was a slight decrease in the noise signal when measured in vacuum (Figure 6b).

**Figure 7:**
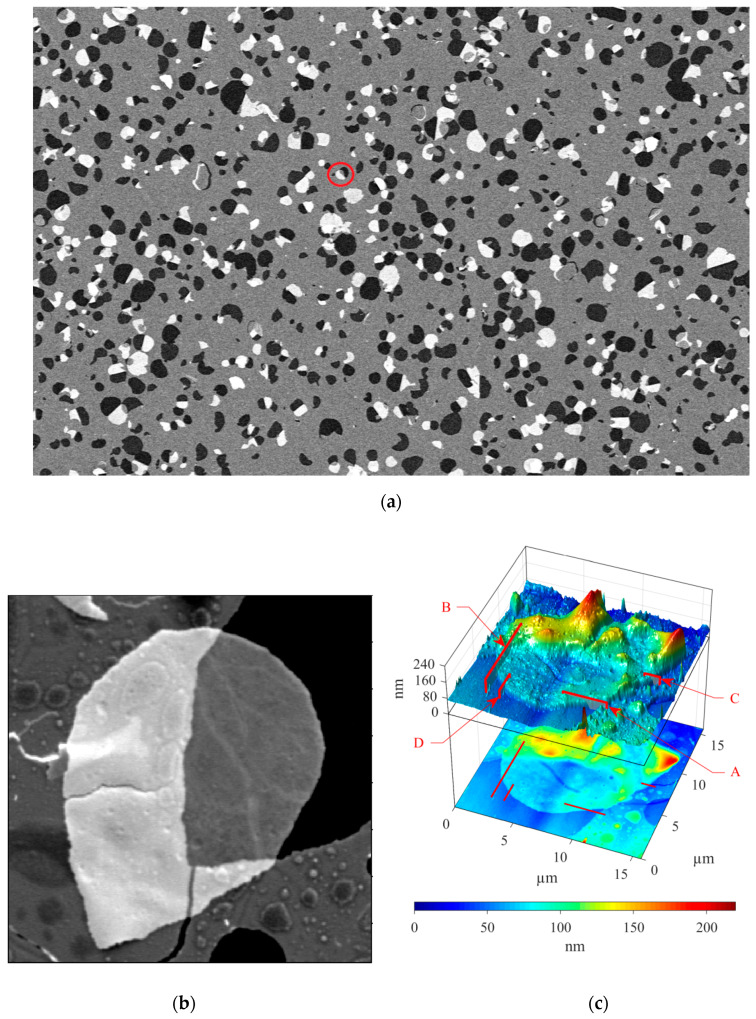

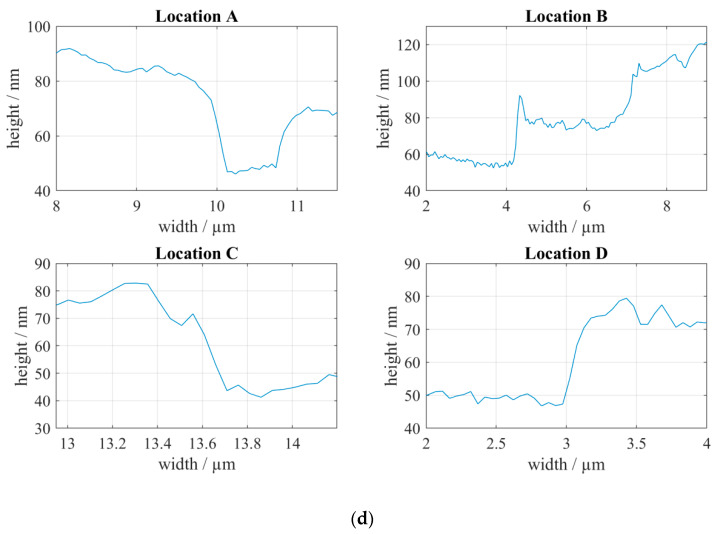
SEM and AFM of the detached portion of the film held on the surface of the sample by electrostatic interaction: (**a**) large area SEM image; (**b**) detailed SEM image; (**c**) AFM image of the region of interest; (**d**) profiles of the chosen areas of the AFM image.

At certain values of the electron beam parameters, a thin film of the material can be transparent (Figure 7). In the images presented in this work (Figure 7a,b), under the transparent film the topography elements of the lower layers were visible. AFM makes it possible to determine the height of the transparent (under the given SEM conditions) nanoscale layer (Figure 7c). The high sensitivity of the AFM in vacuum allows us to accurately determine the height of the material located on the substrate (Figure 7d).

## 4. Discussion

HOPG can be considered as graphene layers connected by van der Waals forces. Graphene, in turn, is known as a material capable of retaining substances in a gaseous state. The problem of the adsorption of ferrocene compounds on the HOPG surface was theoretically and practically considered in [40], where the increased adsorption of molecules resulting from redox reactions on surfaces exposed to atmospheric conditions was confirmed. In the case of the experiment described in this work, adsorption should also be enhanced due to the presence of ozone and surface defectation by the intercalated bismuth atoms. This leads to swelling as a result of gas aggregation under the growing film layer. The carbon oxide on the surface of the HOPG in the defective areas also contributes to the swelling and delamination of the film.

The combination of SEM and AFM is useful in many ways. SEM allows for the precise navigation of the AFM tip on the surface and a constant tip quality control as well. Because the same areas are measured by two techniques, the overlapping of the results provides better material and topographical information than just one method on its own. This makes the AFM and SEM combination an ideal measurement tool for the evaluation of nanomaterials, especially carbon-based nanostructures [41].

When in proximity to the measured sample, the probe can be affected by a number of forces—mechanical contact force, van der Waals forces, capillary forces, electrostatic and magnetic forces, Casimir forces, chemical bonding, solvation forces, etc.—depending on the setup of the microscope and the probe [42,43,44]. These probes consist of a silicon fork and a tip. Alternating current is used to induce vibrations in the fork on its resonance frequency. This allows the probe to function as a detector as well, as any changes in the frequency after calibration show the change in forces affecting the probe and therefore the difference in the sample topography. In the case of this paper, semi-contact mode has been used. A probe is kept in such a distance from the surface that during the vibration of the cantilever it comes into brief contact with the surface [45]. Due to lateral and capillary forces lower than other measurement modes, the semi-contact mode reaches higher resolutions while being more stable and friendlier to the sample as well.

The AFM measurements show dome-like structures and holes on the sample. When the topography imaging by AFM is merged with an SEM material contrast, a distinct difference between the holes and the surrounding material can be seen, as well as the height and thickness of the film. The formation of these bubbles can be explained as a local delamination caused by the compensation of surface tension created by the differing crystal conformation of the substrate and the thin film [46]. The observed swelling is a consequence of compressive mechanical stress as well as the presence of gas products, which can form even more in a vacuum. Kinks and cracks arise as a result of tensile deformations.

In this work, we assume only spherical elements of a swollen film. The pressure value in the chamber was controlled by the electronics and software of the SEM microscope during the measurements in vacuum. Incertitude in the calculations could occur because the atmospheric pressure inside the clean laboratory room could be higher and add positive pressure from 2.5 up to 12.5 Pa. Even the pressure inside the one room could have a differential range from 0.025 to 0.175 Pa [47]. We did not consider these pressure differentials and used literature-based standard atmospheric pressure [48,49]. Knowing the pressure difference and the change in the radius of the film, the following values can be calculated using the following equations.

According to [50], swelling can be calculated by Equation (4):

S = (V − V_0_)/V_0_, (4)

where V_0_ in our case is the volume under normal pressure and V is the volume in vacuum.

The swollen element has the shape of half an ellipsoid, the volume of which can be calculated as half the volume of an ellipsoid by Equation (5):

V = (1/2) (4/3) π·a·b·c,(5)

where a, b and c are the radii of the ellipsoid. In the process of evacuating the vacuum, only one radius changes (denoted by a1); the other two characterize the circle on which half of the ellipsoid (b, c) rises. For location A in Figure 6, the swelling is S = 0.52.

Using data on the height of the tension (swelling) in vacuum, we can calculate the surface tension. Inside the bubbles there is excess pressure, which is compensated by the action of surface tension forces.

The excess pressure under the surface of half an ellipsoid can be described as in Equation (6):

Δp = 2σ/a.(6)

The total pressure exerted on the film consists of the pressure of the medium and the excess pressure of the gases inside. Comparing the total pressure in vacuum and at normal pressure we get Equation (7):

10^−2^ Pa + 2·σ/a = 101,325 Pa + 2*σ/a1. (7)

Substituting location a Figure 6b, the surface tension σ is equal to 6.4 mN/m.

Based on the available data, one can also calculate other parameters, for example, the change in potential energy of the surface molecules by Equation (8):

ΔE = σ·ΔS.(8)

In Equation (8), the area of the layer ΔS is 0.65 µm^2^ and the calculated excess of potential energy ΔE is 4,2 fJ. The observed phenomena were reversible when the “bubbles” maintained their ellipsoid shape. However, some blisters were broken after the evacuation of the chamber (Figure 7). Upon the rupture of the swelling region, a hole is formed, which modifies the surface of the graphite substrate. Bubble rupture is associated with both internal factors (the amount of gas contained in them and their shape) as well as external ones (pressure and temperature [50]). This expansion is limited to height only, however, and does not increase the width of the bubble, which supports the case for this expansion being a result of pressure change. A rupture occurs as a result of local stress exceeding the elastic ability of the thin film to compensate, resulting in the formation of the hole and exposure of the material beneath.

The presented SEM-AFM measurements should be performed after careful adjustment of AFM and tests on various shape calibration samples, because the height differences can be caused by other possible contributions. Among them are ambient conditions as temperature, humidity and the gas composition of the atmosphere. AFM measurements in dry atmospheres (nitrogen, oxygen or argon) could also bring additional information about the chemistry and physics behind the blisters’ formation. The influence of atmosphere conditions could be complex. So, for example, capillary adhesion and friction which depend on humidity will have a different influence on hydrophobic and hydrophilic surfaces [51]. The interplay between the tip and the sample surface also depends on the asperity of a surface morphology. The intensive analysis of such structures under the variation of measurement parameters will contribute to the research of the tip-surface interaction.

## 5. Conclusions

In this work, the results of studying the exfoliation of Bi-Fe-O film from the HOPG surface are presented. An increased interest in multiferroics led the researchers to investigate their combination with other prospective materials. The results aim to fill the space in the field of bismuth ferrite preparation on graphite substrates. The observed film delamination effect can be used to further obtain 2D nano-dimensional materials by the mechanical (for example, ultrasonic) collection of exfoliated flakes.

The results show that simultaneous SEM-AFM contributes to obtaining structural information from local regions as morphological, geometric, physical and chemical characteristics. This is important for a better understanding of the many aspects of material science, such as defects, phase transformations, etc., and the correct interpretation of surface topographic features, as well as its functional role in the testing of the surface in relation to material performance and lifetime.
